# High-Throughput DNA Extraction Using Robotic Automation (RoboCTAB) for Large-Scale Genotyping

**DOI:** 10.3390/plants14152263

**Published:** 2025-07-23

**Authors:** Vincent-Thomas Boucher St-Amour, Vipin Tomar, François Belzile

**Affiliations:** 1 Institut de Biologie Intégrative et des Systèmes (IBIS), Université Laval, Quebec City, QC G1V 0A6, Canada; thomas.st-amour.1@ulaval.ca; 2 Department of Plant Science, Université Laval, Quebec City, QC G1V 0A6, Canada

**Keywords:** high-throughput DNA extraction, RoboCTAB, radicles, genotyping, GBS, Opentrons OT2

## Abstract

Efficient and consistent DNA extraction is crucial for genotyping but often hindered by the limitations of traditional manual processes, which are labour-intensive, error-prone, and costly. We introduce a semi-automated, robotic-assisted DNA extraction (RoboCTAB) tailored for large-scale plant genotyping, leveraging advanced yet affordable liquid-handling robotic systems. The protocol/workflow integrates a CTAB extraction protocol specifically adapted for a robotic liquid-handling system, making it compatible with high-throughput genotyping techniques such as SNP genotyping and sequencing. Various plant parts (leaves, roots, manual seed chip) were explored as the source material for DNA extractions, with the aim of identifying the tissue best suited for collection on a large scale. Young roots (radicle) proved the easiest to harvest at scale, while the harvest of leaves and seed chips were more laborious and error-prone. DNA yield and quality from both leaves and roots (but not seed chips) were similar and sufficient for downstream analysis. Interestingly, root tissue could still be extracted from imbibed seeds, even if the seeds failed to germinate, thus proving useful for DNA extraction. Cost analysis indicates significant savings in labour costs, highlighting the approach’s suitability for large-scale projects. Quality assessments demonstrate that the robotic process yields high-quality DNA, maintaining integrity for downstream applications. This semi-automated DNA extraction system represents a scalable, reliable solution for large-scale genotyping that is accessible to many users who cannot implement highly sophisticated and costly systems as are known to exist in large multinational seed companies. RoboCTAB, a low-cost, optimized method for high-throughput DNA extraction, minimizes the risk of cross-contamination. RoboCTAB is capable of processing up to four 96-well plates (384 samples) simultaneously in a single run, improving cost-efficiency and providing seamless integration with laboratory workflows, potentially setting new standards for efficiency and quality in DNA processing and sequencing at scale.

## 1. Introduction

Since the first DNA extraction by Friedrich Miescher in 1869, DNA extraction is routinely conducted to enable numerous molecular analyses such as PCR amplification, SNP genotyping, and next-generation sequencing. In turn, these technologies enable the generation of extensive genomic information for model, non-model, and underutilized plants and crops. These techniques have greatly enhanced our knowledge of diverse phenomena, i.e., genome structure, function, and phylogenetics [1,2]. While DNA sequencing costs have seen an exponential decline over the last two decades, there has been rather limited progress in terms of the methods used to extract DNA from plants to initiate such molecular analyses outside of very large multinational seed companies using highly sophisticated, expensive, and custom-designed instruments to allow them to handle millions of plant samples [3]. On the scale of thousands to tens of thousands of samples, automated liquid handlers (“robots”) became attractive but have, until relatively recently, been rather expensive. The development of highly affordable liquid handling devices, such as the OT2 system, addresses this challenge. Therefore, most research laboratories face limitations in time and resources, making large-scale DNA extractions challenging. Key to these high-throughput approaches are efficient tissue/explant collection procedures as well as significant automation of the ensuing DNA extraction process.

Leaves or leaf parts remain, by far, the most commonly reported tissue used for extraction of plant DNA, whether it be in the form of leaf punches, leaf segments, or entire young leaves or leaflets [4,5]. Typically, these will be collected either from field- or greenhouse-grown plants or from young plants grown in a controlled environment specifically for the purpose of collecting such samples. While these procedures are feasible, they are time-consuming, labour-intensive, and subject to human error. Other plant tissues such as roots and seeds represent alternative sources of DNA. While seeds or parts of a seed (a so-called “seed chip”) could be seen as an ideal source of plant cells from which to extract DNA as they are typically small, easily compartmentalized (e.g., in multi-well trays), and do not need to be viable, they are also often difficult to grind efficiently and frequently contain large amounts of lipids or carbohydrates that can prove problematic during DNA extraction [6]. Finally, root tissues have been used but less widely than the other two plant parts. These offer many attractive features: (1) root tissue is often the first to emerge from a germinating seed; (2) it is easy to grind as it is not highly lignified; (3) it can be dried or lyophilized very quickly to ensure stability at room temperature; and (4) its cells are more similar to those of leaves in terms of composition (compared to seed).

As for the DNA extraction process, the most commonly used protocols to extract DNA suitable for a multitude of enzymatic modifications (digestion, ligation, amplification, sequencing) are either CTAB-based [7] or rely on the use of commercial purification kits (e.g., DNA capture/purification on a column or magnetic beads). Although the latter typically yield high purity DNA, the per-sample cost of such methods is often in the range of USD 5–10, a cost which rapidly proves prohibitive on a large scale. While CTAB-based protocols offer a good balance between DNA quality/purity and cost, they remain demanding in labour. Advances facilitating the processing of multiple samples such as the use of 96-well plates or blocks, bead grinding, and multichannel pipettors have made it easier to handle hundreds of samples. It nonetheless remains that much of the DNA extraction process requires human labour to do most of the various liquid handling steps that are required in such multi-well extraction procedures. There are ample opportunities for both human error, contamination, and injury (due to repetitive pipetting).

In the present work, we tried to address the growing need for a fast, adaptable, efficient, and largely automated DNA extraction procedure that relies on both a highly efficient explant collection method and a semi-automated DNA extraction protocol (‘RoboCTAB’) executed with the help of a highly affordable liquid handling device (OT2 system by Opentrons https://opentrons.com/robots/ot-2 accessed on 1 September 2023). In what follows, we compare the suitability and efficiency of different plant parts as source materials for DNA extraction and describe a modified CTAB protocol that has been adapted for use on an OT2 system. We determine the success of the DNA extractions by measuring DNA quantity and assessing the suitability of the extracted DNA for use in producing and sequencing GBS libraries. We find that young root tissue (radicle) represents a highly suitable source tissue, and that a widely used CTAB-based protocol can be successfully implemented on an OT2 system. Together, these innovations allow for high-throughput, low-cost, and high-quality DNA extractions that are suitable for next-generation genotyping and sequencing work.

## 2. Results and Discussion

### 2.1. Manual vs. RoboCTAB

In an initial experiment, we aimed to assess the effectiveness of a semi-automated protocol implemented on an Opentrons OT2 liquid handler (RoboCTAB) compared to the standard manual method in terms of DNA yield and quality. To do so, we sampled leaves from 32 soybean seedlings from a single cultivar (Williams 82), half of which were processed using the manual protocol, while the other half was subjected to the RoboCTAB protocol. Both extractions were conducted simultaneously, using identical reagents. The average DNA yield per sample was significantly higher (*p* = 0.004) and almost twice as high using the automated protocol (1.87 µg ± 0.22 vs. 1.06 µg ± 0.13). As a test of suitability for next-generation library construction and sequencing, these DNAs were used to produce GTA genotyping libraries. The average number of sequencing reads per sample was not statistically different (1.55 M ± 0.19 for manual and 1.18 M ± 0.21 for RoboCTAB; *p* = 0.2) (Table 1). Thus, in a direct comparison, the RoboCTAB protocol yielded more DNA than the manual protocol, and the resulting DNA was equally suitable for library construction and sequencing.

### 2.2. Comparing Source Materials for DNA Extraction

Once we had established that the DNA extraction process could be at least partially automated using the OT2 robot, another hurdle was identified in view of enabling high-throughput genotyping—the collection of plant tissues for DNA extraction. While leaves (in whole, in part, or punches) are very widely used, they can be challenging to collect in a timely and error-free fashion. We thus wanted to explore alternative plant parts as source material for DNA extraction. The choice of an optimal process can be thought of as having two components: (1) how easy is it to collect the plant part? and (2) are the DNAs obtained in sufficient quantity and of suitable quality for downstream use?

### 2.3. Harvesting Plant Parts

First, we will consider the collection of plant parts. Starting from a seed, one can imagine cutting off part of the seed (not containing the embryo, for example, as when using industrial seed chippers), collecting young roots that emerge from freshly germinated seeds or young leaf tissue after the germination process has led to a young seedling. While some dry seeds are fairly hard to cut (without harming oneself) depending on the size and shape of the seed (e.g., elongated cereal seeds vs. round soybean seeds), imbibed seeds (after 48 h of imbibition) are much easier to process. Nonetheless, each seed needs to be manually processed—picking the seed from a well in which imbibition was carried out, cutting it, depositing the seed fragment in a new well in an extraction plate—all of this without making mistakes. Roots can be handled in a similar fashion as imbibed seeds, although we found that collecting a portion of the emerged root was much easier and quicker. Also, recently developed devices have been custom-designed to facilitate the harvest of root tissue in a single operation in a 96-well format (SMARTtray; https://smarttray.eu/ accessed on 1 March 2025). Even in the case of seed that had failed to germinate, the seed could be cracked opened to collect a portion of root already present in seeds. Finally, the collection of leaf tissue, despite it being the most commonly used, was the process that proved most problematic. When grown in trays, even fairly young seedlings may become difficult to handle separately (especially for dicots where we want to collect a true leaf and not a cotyledon). Thus, overall, collection of root tissue proved to be the easiest and most amenable to plate-level handling.

### 2.4. DNA Extraction and Sequencing

To more extensively evaluate the suitability of DNA extracted from root tissue for high-throughput analysis, including seed that did not germinate, root- and leaf-derived DNAs were compared on a larger scale. In total, DNA was extracted from 67 different soybean cultivars (29 from leaflets and 38 from embryonic roots collected from imbibed seed that had not germinated). As shown in Table 2, DNA yield was slightly but significantly higher in leaflets, (~3.2 ± 0.4 vs. 2.1 ± 0.2; *p* = 0.008)**.** As above, these DNAs (~100 ng) were used to prepare NGLs for sequencing. Root-derived DNA yielded a greater mean number of sequencing reads per sample, but this difference was not statistically significant (1.55 M ± 0.3 vs. 1.02 M ± 0.2; *p* = 0.102).

However, a significant discrepancy was initially observed in read mapping efficiency. Leaflet-derived reads mapped almost entirely to the soybean reference genome, with an average mapping ratio of 0.99. In contrast, radicle-derived reads exhibited lower mapping efficiency (average ratio of 0.58), with substantial variation among samples. This reduced mapping rate was hypothesized to be primarily attributed to bacterial contamination, as a large proportion of unmapped reads aligned with bacterial genomes. The prevention of bacterial contamination was seen as the best way to prevent this undesirable outcome.

### 2.5. Bleach Treatment for Reducing Bacterial Contamination in Radicles

After observing bacterial contamination in radicle-derived DNA, we investigated whether the addition of bleach to the solution used to imbibe seeds could prevent such contamination. Therefore, one soybean cultivar whose seed had resulted in a low ratio of mapped reads compared to total reads, indicating significant bacterial contamination, was selected for these tests. Seeds were imbibed in water with varying commercial bleach concentrations (0%, 5%, 10%, and 50%) before radicle sampling. The untreated control exhibited the lowest DNA yield (0.53 µg) and a similarly low mapping ratio (0.61) (Table 3). In contrast, all bleach treatments resulted in increased DNA yield (0.96–1.01) and significantly improved mapping efficiency (0.89–0.95). These findings indicate that a moderate bleach concentration (10%) effectively reduces bacterial contamination without significantly compromising DNA yield. Also, other than a slight delay in germination (~12 h), we did not notice any impact on seed germination or seedling growth resulting from the bleach treatment. Thus, incorporating a brief bleach pre-treatment into the radicle DNA extraction workflow prevents bacterial contamination and ensures that the vast majority of DNA entering library construction is derived from the plant.

### 2.6. Manual vs. RoboCTAB Cost Analysis

The comparison between manual and RoboCTAB DNA extraction protocols regarding per-sample costs highlights key differences in labour expenses while showing identical costs for consumables (Table 4). Both protocols incur the same costs for consumables (USD 0.14 per sample) and reagents (USD 0.07 per sample), indicating that automation has no impact on the cost of these items. Notably, however, the human labour cost per sample was significantly lower in the RoboCTAB protocol (USD 0.18) relative to the manual method USD 0.55), resulting in total per-sample costs of USD 0.40 and USD 0.77, respectively. This represents a 48% reduction in overall extraction cost per sample. This reduction in labour cost makes the RoboCTAB protocol more cost-effective, while removing risks or human errors and increasing consistency in sample processing for high-throughput applications.

The above results indicate that this protocol offers a high-throughput solution for DNA extraction, significantly reducing operator hands-on time. The protocol can process up to four 96-well plates per run, with tasks such as liquid handling, reagent dispensing, and plate transfers automated to ensure precision and reproducibility. Although certain steps such as tissue grinding, centrifugation, and plate inversion remain manual, they are comparatively minor, as these are all performed at the plate level and not the well level. Moreover, the automation of time-intensive tasks allows the operator to engage in other activities, thereby increasing overall productivity. Under the RoboCTAB protocol, a single operator can reliably process up to 768 samples (eight 96-well plates) within an eight-hour workday, with approximately three hours of active engagement required for initiating and overseeing the extraction process. While the protocol was designed and performed on an OT2 liquid handler, it can also easily be implemented on the more recent Flex liquid handler by Opentrons. Furthermore, for post-extraction operations such as transferring samples from 96-well to 384-well plates, the OT-2 robot provides high precision, ensuring an error-free process that is essential for maintaining data integrity and streamlining downstream workflows. A significant additional advantage is the robot’s capacity to automate sample cherry-picking, selectively transferring specific wells based on a user-defined coordinate file thereby eliminating a task that is both labour-intensive and prone to human error when performed manually. This automation not only saves time but also ensures greater accuracy and traceability in sample selection. These cost-effective and high-throughput features make our protocol an attractive option for many labs seeking to improve efficiency, reduce errors, and lower consumable costs in high-throughput genotyping workflows in low budgets.

## 3. Material and Methods

### 3.1. Collection of Plant Material

To investigate the advantages and disadvantages of various plant parts as sources of DNA, we started from dried seed in a lab-based setting (i.e., not field sampling). To enable tissue collection from seeds, roots or leaves, soybean seeds were either first imbibed in 24-well trays in distilled water for 48 h or planted in peat pellets (Jiffy Products (NB) Ltd., Pokemouche, NB, Canada). Seeds were either “chipped” by cutting off part of the dry seed (~5 mg) using a surgical blade or a single radicle (~4 mg dried) was excised from each seed using tweezers, in a similar fashion a single leaflet of a trifoliate (~3 mg dried) was collected and placed into a 1.2 mL-well of a deep-well 96-well plate (T110-5, Simport Scientific Inc., Québec, QC, Canada). The samples were then left to dry in a Napco 5831 Vacuum Oven (Napco, Houston TX, USA) set to 30 °C for 48 h. Alternatively, seeds sown in peat pellets were placed under LED lighting and allowed to produce a first true leaf, a process that would typically take between 7 and 12 days. A leaf segment (~1 cm) of an unexpanded unifoliate soybean leaf was collected in a 96-well plate using tweezers. The samples were then left to dry in the vacuum oven for 48 h at 30 °C.

### 3.2. Tissue Grinding

For each sample, a 3 mm tungsten grinding bead was added to each well using a bead-dispensing device (LabTie™ Gravity Bead Loader, Bartlesville, OK, USA). To address electrostatic effects and powder volatility issues during grinding, we used the Opentrons OT-2 robot to dispense 50 µL of TE Buffer (Tris EDTA Buffer, St. Louis, MO, USA) into each well. Plates were then sealed using Clear Adhesive PCR Plate Seals, Cat. No. AB0558, 100 sheets (Thermo Fisher Scientific, Waltham, MA, USA). Bead grinding was performed with a TissueLyser, RETSCH MM 400 (Thermo Fisher Scientific, Waltham, MA, USA) and proper tissue disruption was typically achieved after four 60 s cycles at 24 oscillations per second.

### 3.3. DNA Extraction

Manual DNA extraction was performed in the same deep-well plates as above, with all pipetting being conducted with handheld pipettors (single or multi-channel) using a modified CTAB procedure (Supplementary File S1) adapted from [7]. Semi-automated extraction was performed on an Opentrons liquid handling platform. All automated liquid handling steps were executed using a P300 8-channel electronic pipette mounted on the Opentrons OT2 liquid handler. The major steps of the protocol are as follows (details of reagents and solutions can be found in Supplementary File S2):

(1) Place 96-well plates containing samples and tungsten carbide beads, product no. 69997 (Qiagen, Germantown, TN, USA) on the robot’s deck, add 50 µL of TE Buffer to each well, and seal the plates using Clear Adhesive PCR Plate Seals (Thermo Scientific, Cat. No. AB0558, 100 sheets).
(2) Grind on the TissueLyser by completing four cycles of one minute at 24 oscillations per second (more cycles can be performed if insufficient grinding is achieved).
(3) Place plates on deck, and dispense 400 µL of freshly prepared Lysis Buffer to each well.
(4) Seal the plates using T110 Silicone Sealing Mats (Simport Scientific, Beloeil, QC, Canada), secure the mats using a 4” C-Clamp (Benchmark Scientific, Edison, NJ, USA), invert plates 10 times, then remove sealing mats and incubate for 30 min at 65 °C (when DNA yield is a concern, the incubation can be increased up to 60 min with plate inversions every 20 min).
(5) Place plates on deck and add 400 µL of chloroform/isoamyl alcohol (24:1) solution to form an emulsion (to achieve a good emulsion, the robot injects 300 µL of air at the bottom of each tube ten times, generating a stream of bubbles that facilitates mixing).
(6) Centrifuge for 10 min at 6000 rpm to separate the aqueous and organic phases.
(7) Transfer 360 µL of the upper aqueous phase (containing the DNA) to a fresh deep-well plate, to which 295 µL of cold isopropanol is subsequently added for DNA precipitation.
(8) Seal plates with sealing mats or tape, secure plate cover with C-Clamp and manually invert 20 times.
(9) Centrifuge for 10 min (increase centrifugation time to 30 min for increased yield) then remove seal.
(10) Discard isopropanol either by flipping the plates upside down or by using the robot to remove liquid (this option must be set at the beginning of the run).
(11) Add 295 µL of cold 70% ethanol and centrifuge plates for 10 min (increase centrifugation time to increase yield).
(12) Discard the supernatant (manual inversion or pipetting, as above).
(13) Residual ethanol is removed by a 30 min evaporation in a vacuum oven set to 40 °C.
(14) Add 30–50 µL of elution buffer and incubate at 4 °C for 8 h.
(15) Store plates at −20 °C.

**Figure 1:**
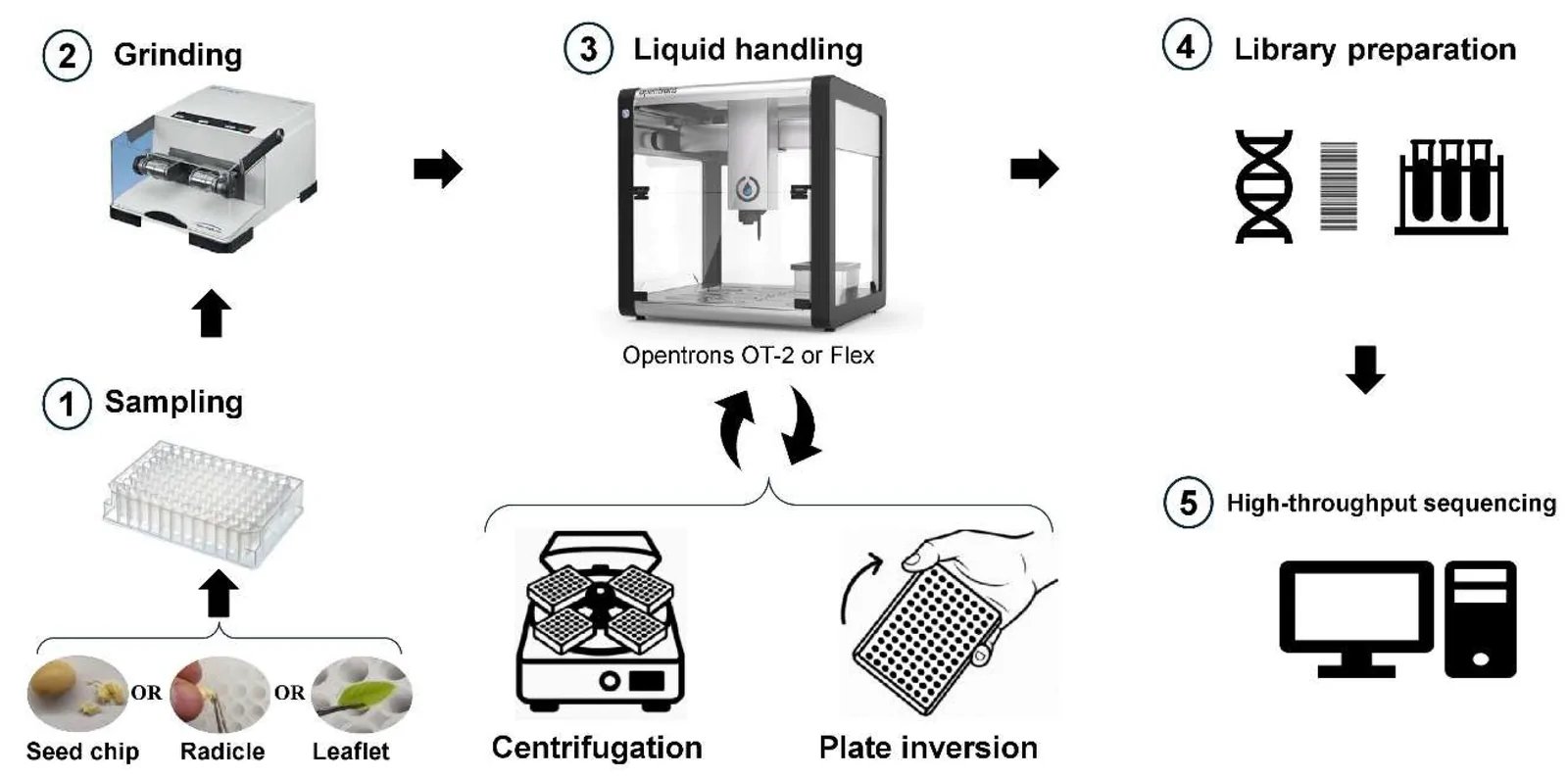
Workflow of high-throughput DNA extraction using RoboCTAB protocol for efficient sample processing. (**1**) Plants are sampled by collecting various plant parts in a 96-well plate. (**2**) Dried plant samples are ground to a fine powder using metallic beads and a mixer mill. (**3**) The DNA extraction protocol is performed, wherein liquid handling steps are performed using an automated liquid handler such as the OT-2 robot, and some additional steps are performed independently (such as centrifugation, incubation, or phase mixing through plate inversion). (**4**) NGS libraries are prepared in 96-, 384- or 1536-well format. (**5**) NGS reads are processed via an appropriate bioinformatic pipeline to extract data from the reads.

**Figure 2:**
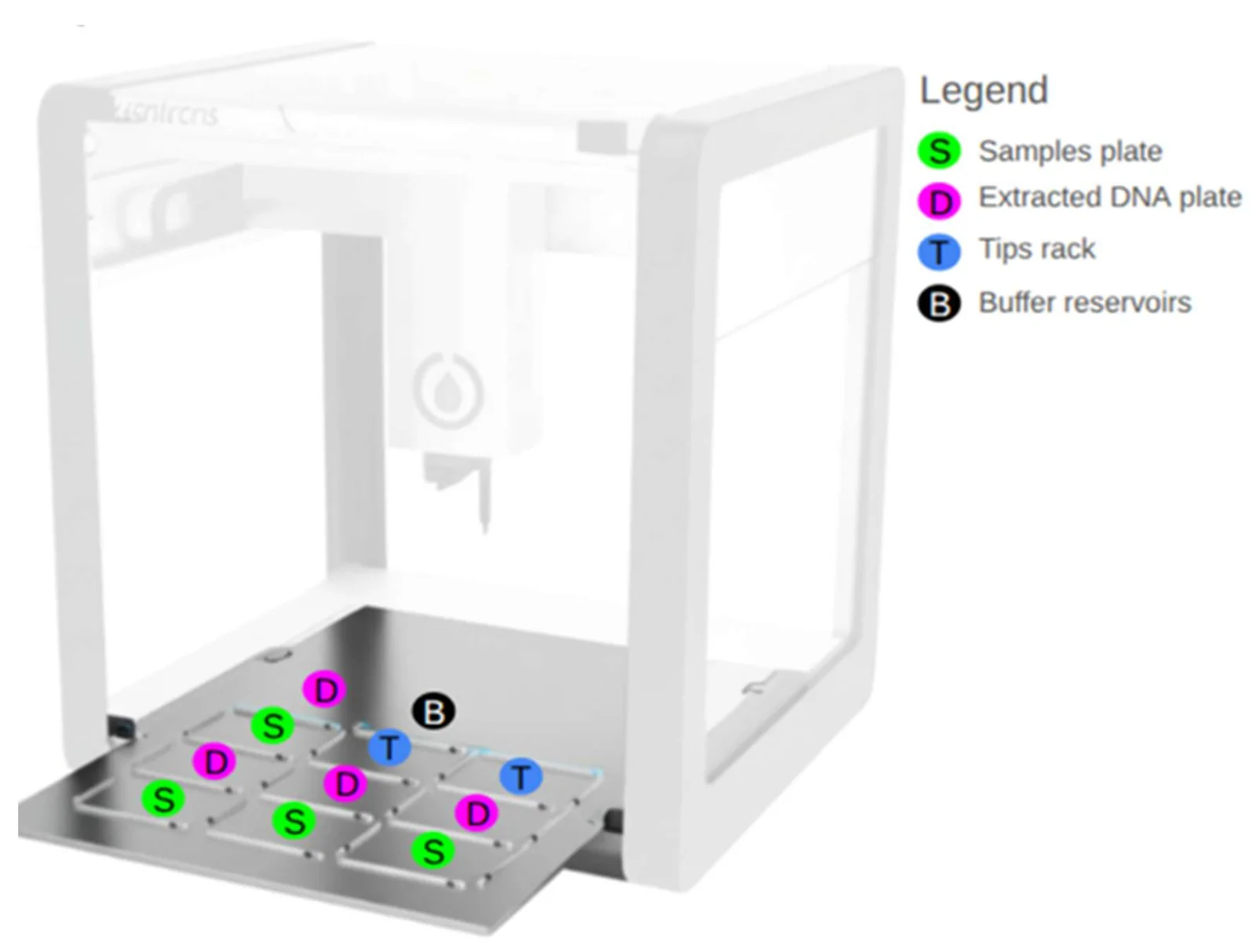
Layout of the Opentrons OT-2 deck and positioning of samples and consumables/supplies for a DNA extraction run of 384 samples with the RoboCTAB protocol.

The programming of the robot was developed using the Opentrons Python API (api level 2.15) and allows the sequential steps of the DNA extraction workflow to be executed. A schematic diagram of the DNA extraction process using the RoboCTAB (Python3, script version 1.0) protocol, illustrating key steps for high-throughput operations, is provided in Figure 1. The script is readily adaptable for processing between 1 and 384 samples per run, leveraging the OT-2 deck’s capacity to hold up to four 96-well source plates simultaneously (Figure 2). The RoboCTAB protocol was developed with an emphasis on user friendliness, providing step-by-step guidance through the Opentrons graphical user interface (GUI) for all stages requiring manual intervention. Depending on the number of samples intended for the extraction run, the script will display on the screen the quantity of reagents that need to be prepared prior to the run.

### 3.4. DNA Quantity and Suitability for High-Throughput Genotyping

The quantification of DNA was performed using the Qubit™ dsDNA HS Assay Kit (Invitrogen, Thermo Fisher Scientific, Waltham, MA, USA) according to the manufacturer’s instructions. To assess the suitability of the extracted DNAs in the context of next-generation genotyping or sequencing, a series of next-generation libraries (NGL) were produced and sequenced. These libraries were produced to assess the performance of the genomic DNAs in various enzymatic processes (digestion, ligation, amplification, and sequencing). NGL#1 comprised 32 samples (soybean leaflets; 16 extracted manually and 16 extracted with the RoboCTAB method). In each case, ~100 ng of each DNA was processed using the GTA genotyping protocol [8], a combination of GBS and amplicon sequencing which yields both genome-wide and gene-/trait-specific SNPs. This library was sequenced on an Element Biosciences AVITI sequencer with 150 bp single-end reads. A second library (NGL#2) was constructed to further assess the quantity and quality of DNA derived from embryonic roots of soybean seed that had failed to germinate. The non-germinating seeds were imbibed for 24 h then the embryonic root (radicle) was collected from 38 such seeds. For comparison, 29 single leaflets were also used to extract DNA. For all 67 samples, 10 µL of DNA (~105 ng/µL for leaflet-derived samples and ~69 ng/µL for radicle-derived samples) was used for GBS library preparation following the original *Ape*KI protocol described by [9]. The resulting GBS library was sequenced for further quality analysis.

## 4. Conclusions

Due to the wide adoption of next-generation sequencing technologies in many realms of research, there is a growing demand for low-cost protocols that can effectively handle large numbers of plant tissues while producing small but sufficient quantities of high-quality genomic DNA. The RoboCTAB protocol described in the present study yielded substantial amounts of genomic DNA from soybean. The quantity and quality of the DNA were adequate to generate GBS-quality DNA, which was then successfully sequenced. Our protocol is highly adaptable, reducing cross-contamination risk allowing for easy scaling to meet experimental requirements. It can perform consistently across a wide range of plant species and process up to four 96-well plates simultaneously, enabling the processing of 384 samples at a time, significantly reducing the labour cost.

## Figures and Tables

**Table 1:** DNA Yield metrics for leaflets between manual and semi-automated (RoboCTAB) method.

Metric	Extraction Method	Mean	SE	*p*-Value
Yield (µg)	Manual	1.06	0.13	0.004
	RoboCTAB	1.87	0.22	
Total Reads (M)	Manual	1.55	0.19	0.2
	RoboCTAB	1.18	0.21	

**Table 2:** DNA yield and sequencing metrics for leaflet and radicle samples.

Metric	Tissue Type	No of Samples	Mean	SE	*p*-Value
DNA Yield (µg)	Leaflet	29	3.2	0.4	0.008
	Radicle	38	2.1	0.2	
Total Reads (M)	Leaflet	29	1.02	0.2	0.102
	Radicle	38	1.55	0.3	
Mapped Reads (M)	Leaflet	29	1.02	0.2	0.03
	Radicle	38	0.61	0.1	

**Table 3:** Effect of a bleach treatment of imbibed seeds on mean DNA yield and mapping efficiency. Each treatment was performed on four seeds.

Bleach Concentration	DNA Yield (µg)	Mapping Ratio
0% (Control)	0.53	0.61
5%	1.01	0.89
10%	0.98	0.95
50%	0.96	0.95

**Table 4:** Cost analysis per sample between Manual and RoboCTAB method.

Spending	Manual	RoboCTAB
	($/per Sample) *	($/per Sample) *
Consumables	0.14	0.14
Reagents	0.07	0.07
Operator time	0.55	0.18
Total cost	0.76	0.39

* Cost calculations were made on the scale of 384 samples.

## Data Availability

RoboCTAB python script is available on https://github.com/tomstamour/RoboCTAB/.

## Supplementary Materials

The following supporting information can be downloaded at https://www.mdpi.com/article/10.3390/plants14152263/s1, File S1: Modified CTAB protocol for manual DNA extraction; File S2: Details of reagents.
