# Supplementary material for: High-Throughput DNA Extraction Using Robotic Automation (RoboCTAB) for Large-Scale Genotyping
Source: Plants (Basel). 2025 Jul 23;14(15):2263. doi: 10.3390/plants14152263 (PMC12348566; doi:10.3390/plants14152263)
Supplement: Supplementary file 1 [file plants-14-02263-s001.zip › plants-3701207-supplementary.docx]

**Supplementary File S1**

**Genomic DNA Extraction with CTAB** Adapted from Doyle, J. J., and J. L. Doyle. (1987). A rapid DNA isolation procedure for small quantities of fresh leaf tissue. Phytochemical Bulletin 19: 11–15

**Manual CTAB DNA extraction protocol (adapted from Doyle and Doyle, 1987) starting from ground tissue:**

1. Shake the Lysis Buffer heated to 65°C well, then add 400 μL to each tube.
2. Invert each plate 20 times and incubate in the water bath at 65°C for 30 minutes, inverting again (20 times) every 10 minutes. *Note:* Open the bottom of the plates before incubation, wrap the plates with rubber bands to prevent them from opening (including the caps), and place a weight to keep them submerged in the water bath.
3. Spin down the plates or centrifuge for 15-20 seconds to bring the liquid to the bottom of the tubes.
4. Place the plates in the freezer/refrigerator for 5 minutes or let them cool at room temperature (optional).
5. Add 400 µL of chloroform/isoamyl alcohol [24:1] under the fume hood.
6. Invert 40 times. Stay under the fume hood, as liquid may spill at this step—chloroform is very volatile, even with closed tubes. Use wooden supports with screws and brown paper on the surface of the tubes to secure them. Place a rubber band over the caps to prevent the contents from leaking.
7. Centrifuge for 10 minutes at 6000 rpm (maximum) using a plate centrifuge with lab support (since chloroform can damage plastic or cause equipment corrosion). If needed, place brown paper over the tubes before centrifuging.
8. Retrieve 200 μL of the supernatant (being careful not to touch the slanted interface) and transfer it to new tubes that are well-labeled and numbered according to the genotype order.
9. Add 160 µL of isopropanol (0.8X the volume of the supernatant).
10. Invert 20 times.
11. Centrifuge for 30 minutes at 6000 rpm.
12. Remove the supernatant, keeping the pellet, and drain on brown paper to remove as much isopropanol as possible. Ensure the pellets remain at the bottom of the tubes—be careful, as the pellet can slip.
13. Add 500 μL of 70% cold ethanol [-20°C] and centrifuge for 30 minutes at 6000 rpm.
14. Remove the ethanol by gently inverting the tube and retain the DNA pellet. Be careful, as the pellet can slip.
15. Drain on brown paper to remove as much ethanol as possible. Briefly centrifuge again and then remove the remaining liquid using a pipette (this can be impractical).
16. Place under a fume hood for 15 minutes (or longer if necessary). Alternatively, place in an incubator at 40°C for 10-15 minutes. Alternate between these two drying methods and remove any residual alcohol with a sterile micropipette. *Note:* Ensure all ethanol has evaporated. Any remaining ethanol may interfere with subsequent enzyme activity and prevent proper sequencing.
17. Suspend the DNA pellet in 50 μL of EB Buffer (Elution Buffer). Add new caps.

**Supplementary File S2**

**Required solutions and buffers:**

1. **5M NaCl:**
   - Dissolve 146 g of NaCl in 400 ml ddH₂O.
   - Adjust the water volume to 500 ml and autoclave for 20 minutes.
2. **1M Tris-HCl, pH 7.5:**
   - Dissolve 60.55 g of Tris in 400 ml ddH₂O.
   - Adjust the pH to 7.5 by adding 32.5 ml of concentrated HCl (at 25°C).
   - Adjust the volume to 500 ml and autoclave for 20 minutes.
3. **Sarkosyl 5%:**
   - Dissolve 35 g of Sarkosyl in 600 ml ddH₂O.
   - Adjust the volume to 700 ml and autoclave for 20 minutes.
4. **Chloroform–isoamyl alcohol (24:1) :**
   - Mix 96 ml of chloroform and 4 ml of isoamyl alcohol.
5. **0.5M EDTA, pH 8.0:**
   - Dissolve 18.61 g of EDTA disodium salt dihydrate to 80 ml ddH₂O.
   - Adjust the pH to 8.0 by adding sodium hydroxide (NaOH) pellets to the solution.
   - Adjust to 100 ml with ddH₂O and autoclave for 20 minutes.
6. **TE Buffer**
   - Mix 5 ml of 1M Tris-HCl, pH 7.5 and 1 ml of 0.5M EDTA, pH 8.0 in 494 ml ddH₂O.
7. **Elution Buffer**
   - Add 5 ml of 1M Tris-HCl pH 7.5 to 490 ml of ddH₂O.
   - Adjust the pH to 8.5 by adding concentrated HCl.
   - Adjust to 500 ml with ddH₂O and autoclave for 20 minutes.
8. **Stock lysis solution A:**
   - 0.35 M Sorbitol: 15.95 g sorbitol
   - 0.1 M Tris-HCl, pH 7.5: 25 ml 1M Tris-HCl, pH 7.5
   - 5 mM EDTA: 2.5 ml 0.5M EDTA, pH 8
   - Adjust to 250 ml with ddH₂O and autoclave for 20 minutes.
9. **Stock lysis solution B:**
   - 0.2 M Tris-Hcl, pH 7.5: 50 ml 1M Tris-HCl, pH 7.5
   - 0.05 M EDTA: 25 ml 0.5M EDTA, pH 8
   - 2M NaCl: 100 ml 5M NaCl
   - 2% CTAB: 5 g CTAB
   - Adjust to 250 ml with ddH₂O and autoclave for 20 minutes.

**10. Preparation of Lysis Buffer (216 ml for four 96-well plates):**

- In a bottle add 90 ml of Stock lysis solution A and 90 ml of Stock lysis solution B.
- Add 36 ml of 5% Sarkosyl.
- Dissolve 1.08 g of sodium metabisulfite (final concentration 0.5%).
- Add 2.16 g of Polyvinyl (PVP MW:40,000) PVPK29-32 (final concentration 1%).
- Mix and place in a water bath set to 65°C.
- Add 108 μL of RNase A to the mixture and homogenize, keeping it at 65°C. (*Note:* Add RNase just before distributing the Lysis Buffer to the ground samples in the plates).
